# Epidemiological, virological and clinical characteristics of HBV infection in 223 HIV co-infected patients: a French multi-centre collaborative study

**DOI:** 10.1186/1743-422X-10-87

**Published:** 2013-03-15

**Authors:** Vincent Thibault, Catherine Gaudy-Graffin, Philippe Colson, Joël Gozlan, Nathalie Schnepf, Pascale Trimoulet, Coralie Pallier, Karine Saune, Michel Branger, Marianne Coste, Francoise Roudot Thoraval

**Affiliations:** 1 Pitie-Salpetriere, Paris, France; 2 Université François Rabelais and CHRU de Tours, Tours, France; 3 Timone University, Marseille-Timone CHU, Marseille, France; 4 Saint Antoine Hospital, Paris, France; 5 Lariboisiere Hospital, Paris, France; 6 CHU Pellegrin, Bordeaux, France; 7 Kremlin Bicetre CHU, Paris, France; 8 Toulouse University Hospital, Toulouse, France; 9 Bichat Hospital, Paris, France; 10 Department of Pharmacy, Nantes CHU, University Hospital of Nantes, Nantes, France; 11 Public Health, H Mondor Hospital, Creteil, France

**Keywords:** Genotype, HBsAg variants, HBV-HIV co-infection, Resistance mutations, Liver lesions

## Abstract

**Background:**

Chronic hepatitis B (CHB) is a clinical concern in human immunodeficiency virus (HIV)-infected individuals due to substantial prevalence, difficulties to treat, and severe liver disease outcome. A large nationwide cross-sectional multicentre analysis of HIV-HBV co-infected patients was designed to describe and identify parameters associated with virological and clinical outcome of CHB in HIV-infected individuals with detectable HBV viremia.

**Methods:**

A multicenter collaborative cross-sectional study was launched in 19 French University hospitals distributed through the country. From January to December 2007, HBV load, genotype, clinical and epidemiological characteristics of 223 HBV-HIV co-infected patients with an HBV replication over 1000 IU/mL were investigated.

**Results:**

Patients were mostly male (82%, mean age 42 years). Genotype distribution (A 52%; E 23.3%; D 16.1%) was linked to risk factors, geographic origin, and co-infection with other hepatitis viruses. This genotypic pattern highlights divergent contamination event timelines by HIV and HBV viruses. Most patients (74.7%) under antiretroviral treatment were receiving a drug with anti-HBV activity, including 47% receiving TDF. Genotypic lamivudine-resistance detected in 26% of the patients was linked to duration of lamivudine exposure, age, CD4 count and HIV load. Resistance to adefovir (rtA181T/V) was detected in 2.7% of patients. Advanced liver lesions were observed in 54% of cases and were associated with an older age and lower CD4 counts but not with viral load or genotype. Immune escape HBsAg variants were seldom detected.

**Conclusions:**

Despite the detection of advanced liver lesions in most patients, few were not receiving anti-HBV drugs and for those treated with the most potent anti-HBV drugs, persistent replication suggested non-optimal adherence. Heterogeneity in HBV strains reflects epidemiological differences that may impact liver disease progression. These findings are strong arguments to further optimize clinical management and to promote vaccination in HIV-infected patients.

## Introduction

Due to common routes of infection, hepatitis B virus (HBV) and immune deficiency virus (HIV) are found concomitantly in approximately 9% of HIV-seropositive patients in Europe [1]. The natural history of HBV infection is complicated in such setting with an increased rate of chronic hepatitis, greater levels of HBV replication and increased incidence of cirrhosis and liver disease-related mortality. Persistence of HBV replication is regarded as a deleterious situation in the setting of HBV-HIV coinfection, and recent guidelines recommend to include, in highly-active antiretroviral therapy (HAART), at least one drug active on HBV and preferentially tenofovir disoproxyl fumarate (TDF) [2-4].

In HBV low-endemic countries several epidemiologic patterns may be identified: patients who lately acquired HBV are usually infected with HBV strains circulating in their living country, whereas patients originating from highly HBV endemic countries are often infected with an HBV strain acquired during childhood in their original country [5,6]. In France, the two main characteristics of HBV infection in the general population are a large diversity of epidemiologic patterns and therefore HBV genotypes, and a low vaccine coverage rate, around 40% [5,7-9]. Since around 7% of HIV-positive patients are infected with HBV, characterization of parameters associated with disease severity and viral resistance is of major importance to optimize therapeutic management [10]. Nevertheless, no nationwide study of HBV genotypic and clinical features in HIV-positive patients has been conducted. A large nationwide cross-sectional multicentre analysis of HIV-HBV co-infected patients was designed to specifically address these issues.

## Materials and methods

### Patients

Ethics statement: As recommended by "la Pitie-Salpetriere Hospital" Institutional Review Board, written information was given to patients for participation to the study. In compliance with the French law regarding clinical studies, when this work was initiated, and since this study was performed on stored samples obtained in the standard of care (no need for any additional blood draw), all patients were provided written information and gave verbal consent. All data were anonymously collected and analyzed. The study was conducted in accordance with the ethics principles of the Declaration of Helsinki. Two hundred and thirty four patients from 19 French hospitals were prospectively included from January to December 2007. Participating centres were distributed in major French regions in order to gather data from a representative sample of patients, independently of their place of living. Inclusion criteria were based on virological parameters. Guidelines regarding HIV and HBV treatment were those reported at that time [11]. Any sample addressed to one of the participating virology laboratories that showed HBV replication over 1000 IU/mL in an HIV-infected patient, whatever the HIV replication level, led to proposition for patient’s inclusion. Only one sample per patient was studied.

### Data collection

Clinical and treatment data were retrospectively collected on patients' clinical files. Final analyzed figures are indicated wherever needed. All data were anonymously collected and analyzed. The study was conducted in accordance with the ethics principles of the Declaration of Helsinki.

### Treatments

A particular attention was made on treatment history for drugs active on HBV replication whether given for treating HIV or HBV infection. Patients were classified as having never received any of these drugs if, in their clinical history, they had not received either interferon (IFN), lamivudine (3TC), emtricitabine (FTC), adefovir (ADV), tenofovir (TDF), telbivudine (LdT) or entecavir (ETV), whatever the indication.

### Virology

HBV viral loads expressed as IU/mL were determined by commercialized standardized techniques. Seventeen laboratories used real-time PCR assays, either Cobas-Taqman with a lower limit of detection (LLOD) of 12 IU/mL (Roche, Meylan, France) or HBV-RealTime with an LLOD of 10 IU/mL (Abbott, Rungis, France), 2 laboratories used either bDNA3.0 with an LLOD of 357 IU/mL (Siemens, Eragny, France) or Cobas-Monitor v2.0 with an LLOD of 60 IU/mL (Roche, Meylan, France).

HBV sequencing methodology was left at investigators discretion but a previously described consensus technical approach, along with a selected set of 37 HBV genotypes A to G Genbank sequences, was distributed and biologists in charge were encouraged to apply this technique to sequence the pol/S genes [12]. Finally, all sequences were centralized to one laboratory in order to confirm the genotyping analysis. Phylogenetic evolutionary analyses were conducted using MEGA-4.0 [13]. In parallel to this approach, all sequences were also submitted to the “HBV-tool” accessible from http://www.hiv-grade.de/cms/grade/hbv-tool.html.

### Statistical analysis

Qualitative data were expressed as numbers and percentages, quantitative data were expressed as mean ± 1 standard deviation or median [1^st^ quartile-3^rd^ quartile], depending on the distribution. Relationships between categorical data were tested by means of the chisquare or Fisher’s exact test when necessary. Means or medians were compared using non parametric tests, Mann Whitney test for two groups, or Kruskal Wallis test for 3 or more groups. To study the independent factors related to an event, a logistic regression analysis was performed, and the adjusted odd ratios (OR) are presented with their 95% confidence intervals. A p value of 0.05 or less was considered significant.

## Results

### Study population

Table 1 describes the main characteristics of the patients included in the study. 223 patients with virological and clinical information forms could be analyzed. Bioclinical data were missing in 1 to 20% of cases, depending on the item. Most patients (178; 80%) were males and mostly of French origin (64.1%) while most women (58.3%) were from African sub-Saharan countries. The main risk factors for male were first, homosexuality (56.8%) followed by intravenous drug use (IVDU, 10.8%) whereas women mostly acquired their infection through heterosexual intercourse (75.6%) and as often as males through IVDU (14.6%).

**Table 1:** Patient's characteristics according to HBV genotype

	**Overall**	**HBV Genotype**				
		**A**	**D**	**E**	**G**	***p value***
**Total number**	223	116	36	52	15	
**Male sex , n (%)**	178 (81)	106 (91)	27 (77)	29 (58)	14 (93)	*<0.001*
**Mean age (sd)**	41.9 (9.0)	43.4 (8.6)	43.5 (5.7)	37.2 (10.0)	44.1 (4.9)	*<0.001*
***Country of Origin***						
Europe (107)	107	74 (77.1)	20 (74.1)	4 (9.3)	9 (69.2)	
North Africa (17)	17	9 (9.4)	4 (14.8)	0	4 (30.8)	
Subsaharan Africa (51)	51	10 (10.4)	2 (7.4)	39 (90.7)	0	*<0.001*
Asia (2)	5	1 (1.0)	1 (3.7)	0	0	
Other (2)	2	2 (2.1)	0	0	0	
***Source of infection***						
Mother-to-child	4	11.0	0	36.4	0	
Homosexual	85	69 (71.9)	5 (15.2)	1 (2.1)	9 (81.8)	
IVDU	22	1 (1.0)	20 (60.6)	1 (2.1)	0	*<0.001*
Heterosexual	73	20 (20.8)	8 (24.2)	41 (87.5)	2 (18.2)	
Other/unknown	6	5 (5.2)	0	1 (2.1)	0	
***Hepatic disease***						
ALT : med [IQR]	43 (29–64)	44.5 (30–64)	41.5 (27–99)	38.0 (28–53)	41.5 (38–92)	*0.39*
Metavir A >1	31/83 (37.3)	14 (35.9)	6 (42.9)	6 (28.6)	4 (50)	*0.70*
Metavir F ≥2	51/94 (54.3)	26 (57.8)	9 (60.0)	8 (34.8)	7 (77.8)	*0.11*
Cirrhosis	17/94 (18.1)	9/45 (20)	2/15 (13.3)	4/23 (17.4)	2/9 (22.2)	*0.9*
***Viral markers***						
HBe Ag + (%)	127/192 (66.1)	75 (78.9)	16 (48.5)	22 (45.8)	11 (91.7)	*<0.001*
Anti HBe + (%)	56/192 (29.2)	18 (18.9)	13 (39.4)	24 (50)	0	*<0.001*
HBV VL log IU/mL	5.26 (3.91–7.4)	5.63 (4.2–7.6)	5.25 (3.7–7.7)	4.97 (3.8–6.5)	3.96 (3.3–7.3)	*0.31*
% with HBV VL > 2000 IU/mL	195 (87.4)	101 (87.1)	31 (86.1)	47 (90.4)	12 (80.0)	0.081
HCV + (%)	28/207 (13.5)	7 (6.5)	19 (54.3)	0	2 (14.3)	<0.001
HDV + (%)	13/164 (7.9)	2 (2.4)	5 (21.7)	5 (11.1)	1 (7.7)	0.011

(Unfrequent genotypes, 1 genotype B, 2 genotypes C and 1 genotype F, were not included in this analysis).

### Treatments

Treatment information was available for all included patients. Most (n = 180, 80.7%) patients benefited or had previously benefited from an anti-retroviral treatment. Among them, past or current HAART included an anti-HBV active drug in 175 (97.2%) patients. However, 106/223 (47.5%) of the patients were not receiving any anti-HBV active antiviral at the time of the study and 17.9% (40/223) had never received any anti-HBV active drug (Table 2). Among those treated with a drug active on HBV (n = 117), 83% (n = 97) were receiving a 3TC or FTC-based treatment either as monotherapy (n = 23, 19.7%) or as combination therapy with TDF in the majority (45/76; 47.9%) of the cases. ADV and ETV were given to 13.7% (16/117) and 1.7% (2/117) of the patients, respectively. Eighteen patients (8.1%) had received or were receiving IFN.

**Table 2:** Main patient's characteristics according to HBV treatment history

	**Never treated**	**Past treatment**	**Currently treated**	**p**
	**(n = 40, 17.9%)**	**(n = 66, 29.6%)**	**(n = 117, 52.5%)**	
**Demographics**				
Sex ratio M/F	26/13	54/11	98/17	0.03
Age m ± sd	38.7 ± 9.3	40.6 ± 10.0	43.8 ± 8.0	0.007
Country of origine				
*Europe*	13 (40.6)	38 (65.5)	57 (60.6)	0.007
*North Africa*	1 (3.1)	2 (3.4)	14 (14.9)	
*Sub-Saharan Africa*	14 (43.8)	17 (29.3)	21 (22.3)	
*Other*	4 (12.5)	1 (1.7)	2 (2.1)	
Source of infection				
*MSM*	10 (29.4)	25 (42.4)	50 (51.0)	
*IVDU*	2 (5.9)	10 (16.9)	10 (10.2)	0.045
*Heterosexual*	19 (55.9)	21 (35.6)	34 (34.7)	
*Other*	3 (8.8)	3 (5.1)	4 (4.1)	
Current HAART	1 (2.5)	37 (56.1)	112 (95.7)	<0.001
**Liver disease**				
*ALT: m ± sd*	56.2 ± 67.3	61.7 ± 68.4	89.2 ± 191	0.15
*Metavir A >1 (83)*	2 (20.0)	10 (43.5)	19 (38.0)	0.43
*Metavir F ≥2 (94)*	9 (64.3)	13 (52.0)	21 (38.2)	0.16
**Viral and immunological markers**				
*HBV VL log UI/mL: m ± sd*	5.0 ± 1.7	5.6 ± 1.9	5.7 ± 1.9	0.16
*HBe Ag +*	22 (59.5)	19 (32.8)	25 (25.0)	<0.001
*HDV+*	1 (2.5)	5 (7.9)	7 (6.1)	0.72
*HCV+*	4 (2.6)	7 (11.5)	18 (16.1)	0.23
*HIV VL log UI: m ± sd*	4.0 ± 1.4	2.9 ± 1.5	2.7 ± 1.5	<0.001
*CD4 count: m ± sd*	398 ± 223	360 ± 266	372 ± 301	0.41
Genotype				
*A*	16 (40.0)	39 (59.1)	61 (52.1)	0.02
*D*	6 (15.0)	9 (13.6)	21 (17.9)	
*E*	16 (40.0)	15 (22.7)	21 (17.9)	
*G*	0 (0)	2 (3.0)	13 (11.1)	
*Other*	2 (4.1)	1 (2.9)	1 (0.7)	
Resistance associated mutations				
*Mutation rt173*	0	6 (9.1)	16 (13.7)	0.04
*Mutation rt180*	1 (2.5)	22 (33.3)	41 (35.0)	<0.001
*Mutation rt181*	0	0	5 (4.3)	0.28
*Mutation rt204*	1 (2.5)	22 (33.3)	35 (29.9)	<0.001

There was a significant propensity for males (85.4%) to benefit from any HBV-active treatment as compared to women (68.3%) (p = 0.018) and treated patients were older than untreated (42.6 vs. 38.7 years; p = 0.03).

Patients from Europe were more often treated (88%) than Sub-Saharan African patients who received less often (73.1%, p = 0.03) anti-HBV drugs.

Among 94 patients with documented liver fibrosis lesions; 5/51 (9.8%) with metavir ≥ F2 were not receiving any treatment at the date of sampling. Conversely, among 17 patients with cirrhosis, only one (5.9%) was not receiving any HBV active treatment at the date of sampling.

### Viral parameters

As expected, HIV viral load was significantly lower in patients receiving ARV treatments and was undetectable by realtime-PCR in 42% (75/180). Surprisingly, anti-retroviral treatment was not associated with a significant difference in median HBV-viral load (HBV-VL) with values of 4.5 log IU/mL [IQR 3.64–7.05] for untreated *versus* 5.6 log IU/mL [IQR 3.97–7.49, p = 0.09] for treated patients. The CD4-cell count was also not significantly different between HAART-treated or -untreated patients and the median values were 397 (IQR 205–606) and 322 (IQR 154–511) cell/mm^3^, respectively (p = 0.24).

Thirteen (7.9%) patients were co infected with HDV and 28 (13.5%) with HCV. Seven patients presented with serological evidence of HCV and HDV co-infection, 5 of them being IVDU.

### HBV Genotypic analysis

#### Genotypes

223 sequences were generated, enabling genotype determination and characterization of drug resistant or HBsAg immune-escape mutants. Genotype distribution (Table 1) shows a vast majority of genotype A (52%) followed by E (23.3%), D (16.1%) and G (6.7%). Data obtained through the phylogenetic approach and the web-based tool were 100% concordant.

Several parameters were directly linked to the genotype. Genotype E was found more often in women (51.2%, p < 0.001) and was significantly associated with heterosexual exposure (87.5%), a younger age (mean 37.2 year, p < 0.001) and a sub-saharan Africa origin (90.9%, p < 0.001). As expected, the prevalence of HBeAg-status was genotype-dependent.

#### HBV resistance mutations

**Figure 1:**
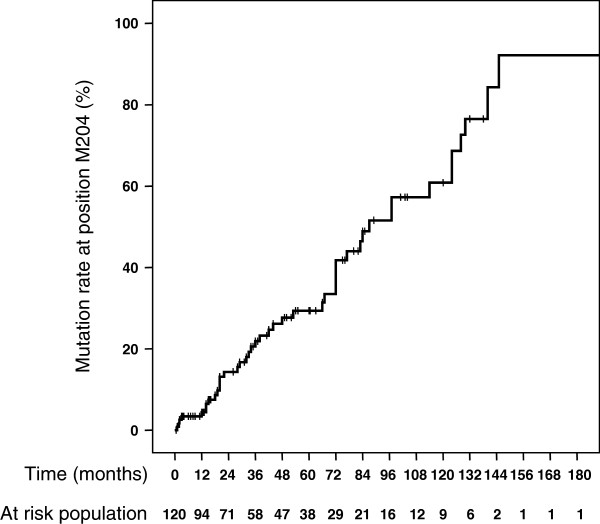
**Cumulative selection of 3TC resistant (rtM204V/I) strains over time in patients who ever received 3TC or FTC.** The number of patients at risk is indicated under the graph. For each patient, the length of 3TC or FTC treatment at the time of sampling was taken into consideration. In half of patients with rtM204 mutation, the duration of treatment was less than 2 years.

All nucleotide sequences (n = 223) were translated into the polymerase and HBs reading frames to analyze for the presence of specific resistant and immune-escape variants (Tables 2 and 3). Previously described resistant mutations i.e. rtV173L, rtL180M, rtA181V/T, rtT184G, rtS202I, rtM204V/I, rtN236T and rtM250V were first studied and then, others residues with changes present in at least two patients were further considered. The most prevalent resistant mutations were rtL180M (28.7%; 64/223) and rtM204V/I (26%; 58/223). Amino-acid change rtM180L was found in 87.5% of the cases associated with rtM204I or V but was found as an isolated mutation in 8 remaining cases. Obviously, resistant-mutations were almost exclusively detected in patients who ever received an antiviral treatment. Overall, only 1 patient who had never received 3TC or FTC for HBV- or HIV-infection presented M204V and L180M mutations. Patients presenting with a 3TC-resistant variant had been treated for a longer period of time as compared to those with a wild-type strain, 44 [20–86] versus 31 [12–60] months, respectively (p = 0.025). Half of patients developed 3TC-resistance mutations (M204-change) less than 2 years after starting 3TC treatment (Figure 1). ADV resistance rtA181T associated mutations were detected in 5 (2.2%) cases; among those, 3 had never received ADV. Noteworthy, all of them had ever received 3TC or FTC. Fourteen patients had received or were receiving ETV but genotypic resistance for this drug was not detected in any patient. HBeAg status was not linked to the development of LAM resistance.

**Table 3:** Main HBs amino-acid changes observed in treated and untreated patients

**HBs amino-acid changes**	**Overall**	**HBV active treatment**			**p**
		**Never**	**Ongoing**	**Stopped**	
**Y100**	10 (4.9%)	1 (2.6%)	5 (4.6%)	4 (7.0%)	0.59
**S114**	113 (52.6%)	16 (41.0%)	60 (53.7%)	37 (58.7%)	0.22
**T118**	6 (2.7%)	1 (2.5%)	4 (3.4%)	1 (1.5%)	0.75
**T125**	27 (12.4%)	4 (10.3%)	15 (13.2%)	8 (12.3%)	0.89
**T126**	3 (1.4%)	0	1 (0.9%)	2 (3.1%)0	0.33
**P127**	71 (32.7%)	19 (50.0%)	34 (29.6%)	18 (28.1%)	*0.04*
**T131**	131 (59.5%)	16 (41%)	75 (64.1%)	40 (62.5%)	*0.03*
**M133**	4 (1.8%)	1 (2.5%)	3 (2.6%)	0	0.427
**Y134**	170 (78.3%)	34 (85.0%)	82 (73.2%)	54 (83.1%)	0.16
**T140**	51 (23.1%)	16 (41.0%)	21 (17.9%)	14 (21.5%)	0.012
**S143**	116 (53%)	15 (38.5%)	63 (55.3%)	38 (59.4%)	0.10
**D144**	5 (2.3%)	2 (5.0%)	3 (2.7%)	0	0.24
**G145**	6 (2.8%)	1 (2.6%)	5 (4.3%)	0	0.25

Absolute number and percentage in parentheses for the considered population are represented.

A multivariate-analysis (Table 4) was conducted to define what parameters were independently associated with the emergence of 3TC resistance. Tested parameters were demographic (age, sex, geographic origin, mode of contamination), 3TC-treatment, HBV-genotype, CD4-count and HIV-viral load. As predicted, 3TC treatment was significantly associated with emergence of resistance mutation but surprisingly, and confirmed by multivariate-analysis, a younger age, lower HIV-viral load and higher CD4-cell count were all protective for the development of 3TC resistance, independently of 3TC treatment.

**Table 4:** Uni- and multivariate analysis on risk factors associated with emergence of rtM204 change

	**rtM204 change**	**Univariate analysis**	**Multivariate analysis**		
**Variable**	**%**	**p**	**OR**	**95% CI**	**p**
**Gender**		0.025			
*men*	29.2				
*Women*	12.2				
**Country of origine**		0.036			
*Europe*	36.4				
*Maghreb*	29.4				
*Sub-Saharan Africa*	15.7				
**3TC or FTC treatment**		<0.001			
*Never*	2.1		1		
*Ever*	31.7		13	1.7–99.2	0.013
	**Presence vs. absence of rtM204 change**				
**Age**	44.8 vs 40.9	<0.001	1.066	1.02–1.11	0.005
**CD4 count**	399 vs 307	0.002	1.001	1.000–1.003	0.038
**T**	1.30 vs 3.41	<0.001	0.503	0.37–0.69	<0.001

Tested variables were: gender, age, geographic origin, mode of contamination, lamivudine treatment, HBV genotype, CD4 cell count, HIV viral load. Analysis was performed on 205 patients among whom 55 carried an rtM204 change.

#### HBsAg variability

Specific mutations within the HBsAg reading frame were sought for, especially those known to influence protein immunogenicity within the major hydrophilic-loop and the a-determinant (Table 3). Amino-acid change sG145R was found in only one patient but sG145A, less frequently described, was detected in 5 sequences (2%). Changes occurring at position s100, 114, 125, 127, 131, 134 140 and 143 were not associated with HBV-treatment and statistics could not be performed for the other residues as changes did not occur at a sufficient frequency. All significant changes (sP127, sT131, sT140) associated with HBV treatment could be explained by genotype-linked polymorphism.

#### Disease severity

Liver disease activity was available for 94 patients as assessed by liver biopsy (56%) or surrogate scores validated in the setting of HIV-HBV co-infection such as Fibrotest® [14]. According to Metavir-classification, 45.7% (43/94) had a mild liver damage while the others were classified with lesions equal to or higher than F2. Patients with more severe liver disease (above F2) were older with a mean age of 43.9 vs. 39.8 years (p = 0.043) and had lower CD4-cell counts with a mean value of 344 vs. 458 cell/mm^3^ (p = 0.046). By contrast, viral load and HBV-genotype were not associated with disease severity. Reliable evaluation of the impact of other hepatitis virus co-infection could not be performed due to missing information on liver histology.

## Discussion

This nationwide collaborative study describes epidemiological, virological, and clinical patterns related to HBV infection in HIV-positive patients with HBV viremia in the era of fully active antiviral treatments in France. Recommendations from international scientific societies have been published and point to an initiation of treatment at a lower HBV-VL than previously recommended [15-18]. Thus nowadays, only very few patients should not be considered for HBV-treatment [19]. Contrary to previous studies, this collaborative work was initiated from a virology laboratory network and patients were randomly selected on the basis of their analytical values.

On a pure technical point of view, our study clearly demonstrates that diffusion of a consensual protocol for HBV-sequencing to multiple laboratories along with a reference sequence library was efficient to generate high quality information regarding HBV genetic variability. We also validated the web-based algorithm proposed on " http://www.hiv-grade.de" that can be largely recommended for any laboratory dealing with HBV sequence.

Unequal distribution of genotypes according to the mode of HBV acquisition has been previously described in a small study from Perez-Olmeda et al. and more recently in the large EuroSIDA study group with comparable conclusions to our study and to recent work in France [5,6,20]. From the genotype distribution and the epidemiological data, identification of very distinct populations may lead to different clinical managements. For instance, genotype D or A infected individuals are most likely IVDU or MSM, respectively, who have certainly acquired both infections either concomitantly or in a short-time interval. By contrast, most HIV-positive women with genotype E HBV infection have likely acquired HBV perinatally and in highly endemic countries. These different sequential events may have a profound impact on the natural history of the disease, particularly since they occur in patients of different gender, age, and immunological status. Very few data are available on the chance to spontaneously clear the virus in these different situations but one may expect very different outcomes in each of them as recently shown in a large US HIV-infected patient cohort [21]. With regards to these observations, it is interesting to note relatively mild liver lesions in genotype E infected patients compared to patients infected with genotype A or D. Thus, natural history of chronic hepatitis B during co-infection should be analyzed with scrutiny as it may largely differ according to the population that is considered and may influence treatment options.

HBV-VL is one of the main criteria to initiate antiviral treatment. In our cohort, HBV-VL was not influenced by HBV genotype but by the presence of HBeAg. Contrary to what observed for HBV mono-infected patients, HBeAg is often present (66%) in HIV co-infected patients, as previously reported [22,23]. Interestingly, HBeAg-negative patients were less often treated, reflecting the current recommendations not to treat patients with low viremia [16].

Among the 15 genotype G infected patients, all but two were HBeAg-positive, which is surprising as genotype G naturally carries the G1896A stop-codon mutation that prevents HBeAg translation from the pre-C start codon [24]. Co-infection with another wild-type viral strain whose genome encodes for HBeAg likely explains a rescue for production of this protein, as reported earlier [25]. Contrary to a recent report from Lacombe et al., we only found a tendency for a more severe liver fibrosis in genotype G infected patients, without statistically significant association [22]. Reasons for this divergence might be due to size differences in the studied populations and also to missing information regarding liver histology in few cases from our study.

One major finding of the present study is the relative low rate of 3TC-resistance mutations. This observation is somewhat surprising as 3TC, or its derivative FTC, is very often a key component in the HAART backbone. As shown on Figure 1 and as previously demonstrated, 3TC treatment induces very rapid emergence of resistance in HIV co-infected patients [26]. The distribution of 3TC-resistance mutations was very similar to what was previously reported even though rtL180M (29.1%) was found more often than rtM204V/I (26%) [27]. Even in previously treated or in currently treated patients, the rate of 3TC-resistance reached only 35%. These data could be explained by a low viral replication before treatment initiation, as low viral replication or an HBe-negative status was shown protective for resistance development in a previous study [28]. The cross-sectional nature of our study did not allow us to collect this information. This relatively low prevalence of resistance is incentive in considering the possibility of low level of compliance to anti-HBV drugs in our cohort.

Adefovir resistance mutations were exceptionally detected (rtA181T-change in 2.2%) and no change was observed for rtN236. Even if one considers that the rtA181T-strains have a slight decreased sensitivity to TDF, it is reassuring to observe a very low prevalence of ADV-resistance, leaving the opportunity to treat these patients with TDF [29]. The rtA181T or -V change is rarely selected by 3TC treatment and one can not exclude that this was not the case for these patients [30,31]. HAART often contains TDF, in addition to FTC or 3TC, a drug that has not been associated with selection of resistant strains so far [32,33]. The use of 3TC in association with TDF in patients from our cohort likely explains the low rate of resistance in patients receiving 3TC and strengthens the benefit of using potent antivirals such as TDF.

Consequence of drug-resistant variant selection on HBs reading frame has been extensively analyzed in our study. Contrary to what has been reported elsewhere, we did not observe significant changes on residues known to affect HBs "a" determinant [34]. In our hands, HBV genotype was the most influencing parameter to explain HBsAg variability and past or current treatment did not seem to particularly affect HBs antigenicity [35]. Key amino-acid changes known to particularly affect HBsAg detection by current immuno-assays or escape vaccination induced antibodies were found in less than 3% of patients [36]. Even though HBV vaccination is less immunogenic in HIV-infected individuals, the low prevalence of immune escape variants in this population should certainly be a strong argument for vaccination campaign to avoid further contamination in at-risk situations [37].

The recognition of significant liver fibrosis in more than half of co-infected and HBV viremic patients in this series, along with a surprising high rate of suboptimal anti-HBV therapy use in this population, point out suboptimal clinical management of HBV infection in HIV-positive patients in France. Such observation was previously reported in several developed countries and highlights the need to promote and improve the education of physicians in charge of HIV-HBV infected individuals [38].

Our study has some limitations due to the recruitment design. It is a transversal study that included patients on the basis of an HBV replication over 1,000 IU/mL whereas no information was collected on the reason for HBV virology assessment. Yet, these patients are clearly those who should benefit from an HBV-antiviral treatment and are those encountered daily in our practice. Noteworthy and contrary to what has been shown by several studies, no direct link was found between liver disease and HBV-VL [39]. This discrepancy might be explained by the relatively low number of patients with complete liver disease assessment in our study or by a different impact of HBV-VL in the context of HIV infection.

In conclusion, our study gives a new insight into the current epidemiology and clinical features of HBV-HIV infection in France. The strong link between the modes of contamination, the contamination event timeline by both viruses and the targeted population suggests that clinical management may be optimized according to this information. Even though potent active drugs with activities against both viruses are available, many patients have not received any anti-HBV treatment despite being treated by HAART and having for half of them advanced liver fibrosis. In addition, persistent HBV viremia in TDF treated patients suggests either non-compliance or suboptimal drug efficacy in this cohort. Finally, the low prevalence of circulating HBsAg immune-escape variants strengthens the need for an active promotion of anti-HBV vaccination of non-immune HIV-infected patients.

## Competing interests

The authors declare that they have no competing interests.

## Authors’ contributions

VT, CGG, PC, JG, NS, PT, CP, KS, MB, MC and the AC11-ANRS members carried out the molecular genetic studies, participated in the sequence alignment and collected patients' clinical information. CGG and VT were in charge of phylogenetic studies and web based analyses. VT and FRT participated in the design of the study and performed the statistical analysis. VT within the AC11-ANRS group conceived the study and coordinated the study. VT, CGG, PC, JG drafted the manuscript. All authors read and approved the final manuscript.
